# Work-Related Psychological Wellbeing and Conservative Christian Belief Among Methodist Circuit Ministers in Britain: Distinguishing Between Emotional Exhaustion and Satisfaction in Ministry

**DOI:** 10.1007/s10943-022-01637-y

**Published:** 2022-09-28

**Authors:** Leslie J. Francis, John M. Haley, Ursula McKenna

**Affiliations:** 1 Centre for Educational Development, Appraisal and Research (CEDAR), University of Warwick, Coventry, UK; 2 World Religions and Education Research Unit, Bishop Grosseteste University, Lincoln, UK; 3 Methodist Circuit Minister (Presbyter), Torbay, UK

**Keywords:** Burnout, Clergy studies, Personality, Empirical theology, Individual differences

## Abstract

Drawing on data provided by 803 Methodist circuit ministers serving in Great Britain, the present study was designed to test the association between conservative Christian belief and work-related psychological wellbeing as operationalised by the balanced affect model proposed by the Francis Burnout Inventory. After taking into account the effects of personal factors, psychological factors, contextual factors, and experience factors, holding conservative Christian belief was associated with a higher level of positive affect (satisfaction in ministry) but independent of negative affect (emotional exhaustion in ministry).

## Introduction

The Methodist Church of Great Britain regards itself as a ‘broad church’, in the sense in which ‘broad church’ has been used to describe the Church of England (Village, 2018). As such the Methodist Church accommodates a wide range of views (Turner, 1998, p. 38) and maintains theological variations within a framework of Christian unity (Hutchings, 1974, pp. 6–7). This accommodation of a wide range of views has sometimes been styled ‘theological pluralism’, but the notion of ‘doctrinal diversity’ may be more fruitful. The notion of doctrinal diversity focuses more closely on ‘the fundamental principles of the historic creeds’ (Methodist Church, 2005, pp. 212–214) and on the authority of scripture. This stance of doctrinal diversity accommodates within the Methodist Church side-by-side ministers who espouse liberal Christian belief and ministers who espouse conservative Christian belief.

Haley and Francis (2006) reported findings from a population survey of circuit ministers serving in the Methodist Church of Great Britain conducted in 1997. A response rate of 74% generated 1,269 completed surveys from ministers serving in itinerant ministry. In terms of mapping the prevalence of liberal Christian belief and conservative Christian belief, this survey reported that 75% of ministers serving in itinerant ministry believed that Jesus physically rose from the dead on the first Easter Sunday, 50% believed in the personal and visible return of Jesus, 34% believed the Bible is the infallible word of God, and 14% believed Christians are in daily conflict with demons.

The same survey was run for a second time in 2008, again as a population study. This time a response rate of 60% generated 874 completed surveys from ministers serving in itinerant ministry. In their comparison of the findings from the two surveys conducted in 1997 and 2008, Haley and Francis (in press) noted that over this period the move was towards a more conservative system of religious belief. While in 1997 three quarters of Methodist ministers believed that Jesus physically rose from the dead on the first Easter Sunday (75%), the proportion rose in 2008 to 83%. While in 1997 a third of ministers believed that the Bible is the infallible word of God (34%), the proportion rose in 2008 to 41%. While in 1997 half of Methodist ministers believed in the personal and visible return of Jesus (50%), the proportion rose in 2008 to 60%. While in 1997 14% of Methodist ministers believed that Christians are in daily conflict with demons, the proportion rose in 2008 to 23%.

A second theme included in the 1997 survey as reported by Haley and Francis (2006) concerned some headline markers of work-related psychological wellbeing. These markers reflected resilience in ministry, satisfaction in ministry, and emotional exhaustion in ministry. In terms of resilience in ministry, 59% of ministers considered that they were successful in overcoming difficulties in ministry. In terms of satisfaction in ministry, 61% of ministers considered that they were accomplishing many worthwhile things in their ministry. In terms of emotional exhaustion in ministry, 45% of ministers considered that they felt emotionally drained by their ministry.

The 2008 replication of the 1997 survey as reported by Haley and Francis (in press) carried these headline markers of work-related psychological wellbeing. In their comparison of the findings from the surveys conducted in 1997 and 2008, Haley and Francis (in press) concluded that the data demonstrated some improvement in work-related psychological wellbeing over this period. The clearest improvement related to the sense of resilience in ministry. While in 1997 59% of Methodist ministers considered that they were successful in overcoming difficulties in ministry, the proportion increased to 67% in 2008. This increase was also reflected in the item concerned with satisfaction in ministry. While in 1997 61% of Methodist ministers considered that they were accomplishing many worthwhile things in their ministry, the proportion rose to 66% in 2008. These increases were mirrored by a decrease in the item concerning emotional exhaustion in ministry. While in 1997 45% of the Methodist ministers considered that they felt emotionally drained by their ministry, the proportion dropped to 42% in 2008.

The analyses undertaken and reported by Haley and Francis (in press) were designed to review these two areas, doctrinal diversity and work-related psychological wellbeing, separately. However, reading these findings side-by-side raises the question regarding potential relationships between the two areas. Might more conservative Christian belief be associated with better work-related psychological health? The present study has been designed to address this question on the data collected in 2008. First, however, it is necessary to examine what is meant by work-related psychological wellbeing among ministers and how this construct is best operationalised. Then it is necessary to consider the other factors that may come into play and potentially contaminate the direct association between conservative Christian belief and work-related psychological wellbeing. Such factors need to be taken into account as control variables.

### Conceptualising and Measuring Clergy Work-Related Psychological Wellbeing

The notion of clergy work-related psychological wellbeing defines a continuum from good wellbeing, through poor wellbeing, to professional burnout. Professional burnout among clergy has been conceptualised and operationalised by two established measures. The longer-established of these two measures, the Maslach Burnout Inventory (Maslach & Jackson, 1986) was designed for generic use within the caring professions and has been applied in a number of studies among clergy, including work reported by Warner and Carter (1984), Crea (1994), Strümfer and Bands (1996), Rodgerson and Piedmont (1998), Stanton-Rich and Iso-Ahola (1998), Virginia (1998), Evers and Tomic (2003), Golden et al. (2004), Raj and Dean (2005), Miner (2007a, 2007b), Doolittle (2007, 2010), Chandler (2009), Joseph et al. (2010), Buys and Rothmann (2010), Parker and Martin (2011), Joseph et al. (2011), Rossetti (2011), Küçüksüleymanoğlu (2013), Rossetti and Rhoades (2013), Herrera et al. (2014), Proeschold-Bell et al. (2014), Crea and Francis (2015), Adams et al. (2017), Büssing et al. (2017), Vicente-Galindo et al. (2017), Case et al. (2020), Malcolm et al. (2021), and Proeschold-Bell et al. (2022).

Rutledge and Francis (2004) suggested that some of the items in the Maslach Burnout Inventory failed to reflect the experience and vocabulary of clergy, and proposed a modified form of this instrument for use among religious leaders. This modified instrument has been tested in a series of studies, including Francis and Rutledge (2000), Kay (2000), Francis et al. (2004), Francis and Turton (2004a, 2004b), Hills et al. (2004), Randall (2004, 2007), Rutledge (2006), Turton and Francis (2007), Francis et al. (2007), Francis et al. (2010), and Miller-Clarkson (2013). However, tight control over copyright has prevented this modified instrument being more widely used by other researchers.

The Maslach Burnout Inventory conceptualised burnout in terms of three components, sequentially related to each other. Emotional exhaustion gives rise to increasing depersonalisation of clients. Depersonalisation gives rise to decreasing sense of personal accomplishment as clients who experience depersonalisation may become less accepting of the service offered to them (Maslach, 2003). Although this theory is attractive, it is difficult to validate empirically. Moreover, the theory is better for diagnosing burnout than for remedial intervention.

The more recent of the two measures, the Francis Burnout Inventory (Francis et al., 2005) was designed specifically for use among religious professionals and clergy and has been employed in a diverse range of studies, including work reported by Francis et al. (2008), Francis et al. (2009), Robbins and Francis (2010), Brewster et al. (2011), Francis et al. (2012), Robbins et al. (2012), Barnard and Curry (2012), Randall (2013a, 2013b, 2015), Francis et al. (2013a, 2013b, 2013c), Robbins and Francis (2014), Francis et al. (2015), Sterland (2015), Francis and Crea (2015), Durkee-Lloyd (2016), Francis and Crea (2018, 2021), Francis et al. (2019a) and Francis et al. (2021b).

The Francis Burnout Inventory, rooted in the work of Bradburn (1969), conceptualises burnout as comprising two components, related one to the other through the model of balanced affect. According to Bradburn’s theory positive affect and negative affect are relatively independent psychological systems, rather than opposite poles of a single continuum. As a consequence it is possible for individuals to record both high levels of negative affect and high levels of positive affect. According to Bradburn’s theory, high levels of positive affect are able to offset the deleterious consequences of high levels of negative affect. According to the balanced affect model of psychological wellbeing, warning signs of poor work-related psychological health occur when *low* levels of positive affect coincide with *high* levels of negative affect. The advantage of this model is that it generates theories about how the problems of burnout or poor work-related psychological health among clergy may be addressed in terms of preventative and remedial strategies. Although it may not be possible to reduce the causes of emotional exhaustion in ministry, it may be possible to compensate for high levels of emotional exhaustion by maximising strategies for enhancing the sense of satisfaction in ministry.

The Francis Burnout Inventory operationalised these two notions of negative affect and positive affect in ways that mapped closely onto the experiences of working clergy: negative affect was operationalised in the Scale of Emotional Exhaustion in Ministry; positive affect was operationalised in the Satisfaction in Ministry Scale. A series of studies has now validated the balanced affect model of clergy work-related psychological wellbeing, including work reported by Francis et al. (2011) among 744 clergy in The Presbyterian Church USA, Francis et al. (2017b) among 658 clergy in the Church of England, Francis et al. (2017c) among 155 priests in the Roman Catholic Church in Italy, Francis et al. (2017a) among 95 priests and 61 religious sisters in the Roman Catholic Church in Italy, Village et al. (2018) among 358 Anglican clergy in the Church in Wales, Francis et al.,(2019b) among 99 Anglican clergy in England, and Francis et al. (2021a) among 287 priests in the Roman Catholic Church in Italy.

### Taking Control Variables Into Account

Studies exploring individual differences in clergy work-related psychological wellbeing have drawn attention to the importance of personal factors, psychological factors, and contextual factors that may be important to take into account before exploring the association between conservative Christian belief and burnout (see Francis, 2018 for an overview). The two personal factors routinely taken into account are sex and age. Although in terms of sex differences there is no consistent finding across studies, there is a consistent finding in relation to age difference. Lower levels of burnout are found among older clergy. What is not so clear, however, is the explanation given for this age effect. It may be explained partly by the suggestion that more experienced and older clergy have learned how to deal better with the stresses of ministry. It may be explained partly by the suggestion that clergy prone to burnout may have left the clerical profession early, and are now absent from the older age group of serving clergy. For this reason, alongside age, studies may wish to include a question concerning the number of years served in ministry.

Concerning psychological factors, by far the most consistent finding concerns the impact of personality on clergy work-related psychological wellbeing (see Francis, 2018). Studies employing the Big Five Factor model of personality (Costa & McCrea, 1985), the Eysenckian three dimensional model of personality (Eysenck & Eysenck, 1991) or the Francis Psychological and Emotional Temperament Type Scales (Village & Francis, 2022) consistently find that good work-related psychological wellbeing is associated with higher extraversion scores and lower emotionality (neuroticism) scores. These two dimensions of personality can be characterised in the following way. Eysenck and Eysenck (1991, p. 4) describe typical introverts as quiet, retiring, introspective, reserved and distant except to close friends. By way of contrast, typical extraverts are described as sociable and talkative, people who like parties, have many friends, and dislike studying by themselves. Eysenck and Eysenck (1991, pp. 4–5) describe higher scorers on the neuroticism scale as anxious, worrying, moody, and frequently depressed. They are overly emotional, react strongly to things, and find it difficult to restore equilibrium after emotionally arousing experiences. Low scorers on the neuroticism scale, by way of contrast, are usually calm, even-tempered, controlled and unworried. They tend to respond emotionally only slowly and generally weakly, and to regain equilibrium quickly.

Concerning contextual variables, two factors that the present study proposes to take into account are marital status (married or not married) and the number of churches within pastoral care.

### Research Question

Against this background, the present study has been designed to assess the association between conservative Christian belief, first with negative affect (emotional exhaustion in ministry) and then with positive affect (satisfaction in ministry), after in each case taking into account the effects of personal factors (sex and age), psychological factors (extraversion and emotionality), experience in ministry (years engaged in circuit ministry and years in present appointment), and contextual factors (marital status and number of churches within pastoral charge).

## Method

### Procedure

The *Methodist Circuit Ministers’ Survey 2008* was distributed by post (during May) to all 1,584 ministers serving in circuit appointments, as published in the *Minutes of the Annual Conference and Directory* for the Methodist Church year 2007–2008. The survey was accompanied by a pre-paid reply envelope. Participants were assured of anonymity and confidentiality. Appropriate follow-up reminder letters resulted in the return of 951 thoroughly completed questionnaires, a response of 60%, among whom there were 803 ministers who self-identified as currently engaged in itinerant stipendiary ministry either as superintendent or ordained minister, and who fully completed the instruments employed in the current analyses.

### Measures

*Work-related psychological wellbeing* was assessed by the two scales proposed by the Francis Burnout Inventory (FBI; Francis et al., 2005). This instrument comprises two 11-item scales, the Scale of Satisfaction in Ministry (SIMS), designed to capture positive affect, and the Scale of Emotional Exhaustion in Ministry (SEEM), designed to capture negative affect. Each item is rated on a five-point Likert scale: disagree strongly (1), disagree (2), not certain (3), agree (4), and agree strongly (5). Example items concerned with satisfaction in ministry are: ‘The ministry here gives real purpose and meaning to my life’ and ‘I gain a lot of personal satisfaction from working with people in my current ministry’. Example items concerned with emotional exhaustion are: ‘I feel drained in fulfilling my ministry roles’ and ‘I am less patient with those among whom I minister than I used to be’. In their foundation paper for the FBI, drawing on a sample of 6,680 clergy from Australia, England, and New Zealand, Francis et al. (2005) reported the following alpha coefficients: SEEM, *α* = 0.84; SIMS, *α* = 0.84.

*Personality* was assessed by the short form of the Eysenck Personality Questionnaire Revised (EPQR-S; Eysenck et al., 1985). This instrument comprises four 12-item scales designed to measure extraversion (E), neuroticism (N), and psychoticism (P), together with a lie scale (L). Each item is rated on a two-point scale: no (0), and yes (1). Example items concerned with extraversion are: ‘Are you a talkative person?’ and ‘Do you like mixing with people?’. Example items concerned with neuroticism are: ‘Does your mood often go up and down?’ and Are your feelings easily hurt?’. Example items concerned with psychoticism are: ‘Do you prefer to go your own way rather than act by the rules?’ and ‘Would you like other people to be afraid of you?’. Example items from the lie scale are: ‘If you say you will do something, do you always keep your promise no matter how inconvenient it might be?’ and ‘Are all your habits good and desirables ones?’. In their foundation paper for the EPQR-S, Eysenck et al. (1985) drawing on samples of 408 males and 494 females (students, teachers, and other willing and varied participants), reported the following alpha coefficients: E, *α* = 0.88 (males) and 0.84 (females); N, *α* = 0.84 (males) and 0.80 (females); P, *α* = 0.62 (males) and 0.61 (females); L, *α* = 0.77 (males) and 0.73 (females).

*Conservative Christian belief* was assessed by the newly proposed Haley Index of Conservative Christian Belief (HICCB), designed for the present study. This six-item instrument focused on beliefs about Jesus and the Bible. Each item is rated on a five-point Likert scale: disagree strongly (1), disagree (2), not certain (3), agree (4), and agree strongly (5). Example items are ‘The Bible is the infallible Word of God’ and ‘Jesus physically rose from the dead on the first Easter Sunday’. In the present study this scale reported an alpha coefficient of 0.89.

*Sex* was recorded as a binary option: male (1) and female (2).

*Age* was recorded within six categories: under 26 (1), 26–35 (2), 36–45 (3), 46–55 (4), 56–65 (5), and over 65 (6).

*Marital status* was recorded as a binary option: married (2), and not married (1).

*Experience in ministry* was assessed by two questions: ‘How long have you been engaged in circuit ministry?’ was rated on a five-point scale: 10 years or less (1), 11–20 years (2), 21–30 years (3), 31–40 (4), and 41 years or more (5); ‘How many years have you been in your present appointment?’ recorded the actual number.

*Environmental context for ministry* was assessed by the question, ‘Total number of churches in your pastoral change (superintendents should not include whole circuit)’ and the actual number was recorded.

### Participants

The 803 participants included in the analysis comprised 558 men and 245 women (Table 1). In terms of age, 23 ministers were between 26 and 35 years, 140 between 36 and 45 years, 306 between 46 and 55 years, 310 between 56 and 65 years, and 24 over the age of 65 years; 681 were married, 121 not married, and one preferred not to say. In terms of experience of circuit ministry, 277 had been engaged in circuit ministry for 10 years or less, 304 for 11–20 years, 149 for 21–30 years, 64 for 31–40 years, and 9 for 41 years or more. In terms of length of time in present appointment, 188 had been in post for one or two years, 227 had been in post three or four years, 170 had been in post for five or six years, 104 had been in post for seven or eight years, 55 had been in post for nine or ten years, 49 had been in post for more than ten years, and 10 preferred not to say. In terms of numbers of churches in their pastoral charge, 96 reported one church, 212 two churches, 208 three churches, 117 four churches, 63 five churches, 41 six churches, 25 seven churches, 34 eight or more churches, and 8 preferred not to say.

**Table 1:** Sample profile

	N
*Sex*	
Male	558
Female	245
*Age*	
26–35	23
36–45	140
46–55	306
56–65	310
Over 65	24
*Status*	
Married	681
Not married	121
Preferred not to say	1
*Time in circuit ministry*	
Up to 10 years	277
11–20 years	304
21–30 years	149
31–40 years	64
Over 40 years	9
*Time in present appointment*	
1–2 years	188
3–4 years	277
5–6 years	170
7–8 years	104
9–10 years	55
Over 10 years	49
Preferred not to say	10
*Churches in pastoral charge*	
1	96
2	212
3	208
4	117
5	63
6	41
7	25
8 +	34
Preferred not to say	8

### Analysis

The data were analysed by the SPSS statistical package, drawing on the frequency, correlation, reliability, and regression routines.

## Results and Discussion

The first step in data analysis explored the scaling properties of the three core instruments employed in the study. Table 2 presents scale properties for the two components of the Francis Burnout Inventory (Scale of Emotional Exhaustion in Ministry and Satisfaction in Ministry Scale) in terms of the correlations of the individual items with the sum of the other ten items, and the item endorsement, combining the agree and agree strongly responses. In respect of the Scale of Emotional Exhaustion in Ministry, these data demonstrate good item homogeneity with the correlations ranging between 0.40 and 0.76 and a good range of items discrimination with endorsement ranging between 14 and 49%. In respect of the Satisfaction in Ministry Scale, these data demonstrate good item homogeneity within the correlations ranging between 0.35 and 0.73 and a good range of item discrimination with endorsement ranging between 45 and 83%.

**Table 2:** Francis Burnout Inventory: Scale properties

	*r*	%
*Scale of Emotional Exhaustion in Ministry*		
I feel drained by fulfilling my ministry roles	0.60	48
Fatigue and irritation are part of my daily experience	0.67	39
I am invaded by sadness I can’t explain	0.55	15
I am feeling negative or cynical about the people with whom I work	0.64	15
I always have enthusiasm for my work*	0.49	49
My humour has a cynical and biting tone	0.40	15
I find myself spending less and less time with those among whom I minister	0.48	41
I have been discouraged by the lack of personal support for me here	0.51	15
I find myself frustrated in my attempts to accomplish tasks important to me	0.57	48
I am less patient with those among whom I minister than I used to be	0.54	26
I am becoming less flexible in my dealings with those among whom I minister	0.53	14
*Satisfaction in Ministry Scale*		
I have accomplished many worthwhile things in my current ministry	0.58	72
I gain a lot of personal satisfaction from working with people in my current ministry	0.65	83
I deal very effectively with the problems of the people in my current ministry	0.50	45
I can easily understand how those among whom I minister feel about things	0.35	61
I feel very positive about my current ministry	0.68	68
I feel that my pastoral ministry has a positive influence on people’s lives	0.47	82
I feel that my teaching ministry has a positive influence on people’s faith	0.36	74
I feel that my ministry is really appreciated by people	0.54	75
I am really glad that I entered the ministry	0.62	82
The ministry here gives real purpose and meaning to my life	0.70	68
I gain a lot of personal satisfaction from fulfilling my ministry roles	0.73	78

*This item has been reverse coded to compute the correlations, but not the percentage endorsement

*r* = correlation between individual items and the sum of the remaining items

% = item endorsement, combining the agree and agree strongly responses

Table 3 presents scale properties for the Haley Index of Conservative Christian Beliefs. Here the data demonstrate good item homogeneity with the correlations ranging between 0.64 and 0.79 and a good range of item discrimination with endorsement ranging between 41 and 94%.

**Table 3:** Haley Index of Conservative Christian Belief: Scale properties

	*r*	%
Jesus fully God and fully human	0.66	94
Jesus died on the cross in atonement for sins	0.74	86
Jesus physically rose from the dead on the first Easter Sunday	0.79	81
Jesus was conceived by the Holy Spirit and born of the Virgin Mary	0.78	78
The miracles of Jesus were historical and literal	0.74	57
The Bible is the infallible Word of God	0.64	41

Table 4 presents the mean scale scores and internal consistency reliability in terms of the alpha coefficient (Cronbach, 1951) for all five measures subsequently employed in the regression models: emotional exhaustion, satisfaction in ministry, conservative belief, and extraversion and neuroticism. All five measures record alpha coefficients in excess of 0.80.

**Table 4:** Mean scale scores and internal consistency reliability

	Alpha	N items	Mean	SD
Emotional exhaustion	0.86	11	29.39	6.87
Satisfaction in ministry	0.87	11	41.56	5.28
Extraversion	0.87	12	6.74	3.64
Neuroticism	0.82	12	4.28	3.15
Conservative belief	0.89	6	23.13	5.05

The second step in data analysis contextualised the mean scale scores recorded by Methodist ministers on the two scales proposed by the Francis Burnout Inventory alongside other studies that have employed this instrument among other groups of clergy. These data are presented in Table 5 in respect of the Scale of Emotional Exhaustion in Ministry and in Table 6 in respect of the Satisfaction in Ministry Scale. In terms of emotional exhaustion in ministry Methodist ministers recorded high scores alongside Church of England clergy. In terms of satisfaction in ministry Methodist ministers recorded low scores alongside Church of England clergy.

**Table 5:** Mean scores of Scale of Emotional Exhaustion in Ministry

	*N*	Mean	*SD*
1. Australian clergywomen^e^	212	24.3	5.9
2. Newfrontiers lead elders^f^	134	25.3	6.9
3. Catholic priests in Italy^h^	155	25.5	6.9
4. Australia, England and New Zealand^b^	3715	26.0	6.5
5. Church of England clergywomen^c^	874	27.6	6.6
6. United States of America^a^	748	27.8	7.9
7. Church in Wales clergymen^g^	266	28.2	7.4
8. Methodist circuit ministers^i^	803	29.4	6.9
9. Church of England clergy^d^	521	29.6	7.4

^a^From Francis et al. (2008)

^b^From Francis et al. (2009)

^c^From Robbins and Francis (2010)

^d^From Brewster et al.(2011)

^e^From Robbins et al. (2012)

^f^From Francis et al. (2012)

^g^From Francis et al.(2013a)

^h^From Francis and Crea (2015)

^i^The present study

**Table 6:** Mean scores of Satisfaction in Ministry Scale

	*N*	Mean	*SD*
1. Newfrontiers lead elders^f^	134	45.2	4.6
2. United States of America^a^	748	44.5	5.7
3. Australian clergywomen^e^	212	44.2	4.5
4. Church of England clergywomen^c^	874	43.7	4.5
5. Australia, England and New Zealand^b^	3715	43.2	4.9
6. Catholic priests in Italy^h^	155	42.6	5.1
7. Church in Wales clergymen^g^	266	42.1	5.1
8. Methodist circuit ministers^i^	803	41.6	5.3
9. Church of England clergy^d^	521	39.5	4.9

^a^From Francis et al. (2008)

^b^From Francis et al. (2009)

^c^From Robbins and Francis (2010)

^d^From Brewster et al.(2011)

^e^From Robbins et al. (2012)

^f^From Francis et al. (2012)

^g^From Francis et al.(2013a)

^h^From Francis and Crea (2015)

^i^The present study

The third step in data analysis explored the bivariate associations between the variables introduced into the present study, namely the two dependent variables (exhaustion in ministry and satisfaction in ministry) and the five sets of predictor variables: personal factors (sex and age), psychological factors (extraversion and neuroticism), contextual factors (married and number of churches), experience in ministry (years in ministry and years in present post), and theological factors (conservative belief). Five features of the correlation matrix presented in Table 7 merit commentary.

**Table 7:** Correlation matrix

	Sex	Age	P	S	C	M	N	E	CB	SIMS
Emotional exhaustion (SEEM)	−0.00	−0.17^***^	−0.06	−0.11^***^	0.00	−0.09^*^	0.56^***^	−0.18^***^	−0.06	−0.66^***^
Satisfaction in ministry (SIMS)	−0.02	0.06	0.09^*^	0.04	−0.08^*^	0.04	−0.42^**^	0.28^***^	0.19^***^	
Conservative belief (CB)	−0.06	−0.12^***^	−0.07^*^	−0.23^***^	0.10^**^	0.04	−0.03	0.11^**^		
Extraversion (E)	0.09^**^	−0.05	0.05	−0.10^**^	−0.07^*^	0.02	0.02			
Neuroticism (N)	0.07	−0.13^***^	−0.06	−0.07^*^	0.00	−0.08^*^				
Married (M)	−0.31^***^	0.04	0.06	0.14^***^	−0.10					
Number of churches (C)	−0.00	0.08^*^	−0.03	−0.05						
Years of service (S)	−0.39^***^	0.47^***^	0.25^***^							
Years in post (P)	−0.12^***^	0.10^**^								
Age	−0.10^**^									

*p < .05, **p < .01, ***p < .001

First, in terms of personal factors, there are seven significant associations with age. Two of these are to be expected: older minsters tend to have recorded more years in service and more years in their present post. Older ministers recorded lower scores of emotional exhaustion and lower scores of neuroticism. This is consistent with other studies that find this trend (Francis, 2018). The trend can be explained by the suggestion that younger more emotionally labile and emotionally exhausted ministers may have opted out of ministry before reaching the older age group. The data also demonstrate that older ministers are less likely to hold conservative belief and have been given charge of more churches. The negative correlation between sex and age demonstrate that the average age of female ministers is lower than the average age of male ministers. The other four significant correlations with sex confirm that female ministers have recorded fewer years of overall service and fewer years in their present post, that female ministers are less likely than male ministers to be married, and that female ministers tend to be more extravert than male ministers.

Second, in terms of psychological factors, both extraversion and neuroticism emerged as strong predictors of individual differences in both emotional exhaustion and satisfaction in ministry. Higher levels of emotional exhaustion are associated with neurotic introversion, while higher levels of satisfaction are associated with stable extraversion. These findings are consistent with the wider literature associating personality and positive psychology (Francis, 1999). Higher levels of conservative belief are associated with higher extraversion scores, but are independent of neuroticism scores.

Third, in terms of contextual factors, being married is correlated with lower emotional exhaustion scores, but independent of satisfaction in ministry scores. Having responsibility for more churches is correlated with lower satisfaction in ministry scores, but independent of emotional exhaustion scores.

Fourth, in terms of experience factors, the number of years in service (like age) is correlated with lower levels of emotional exhaustion, but independent of satisfaction in ministry. The pattern is different, however, for years in present post. The number of years in present post is correlated with higher levels of satisfaction in ministry, but independent of emotional exhaustion.

Fifth, higher levels of conservative belief are correlated with greater satisfaction in ministry, but independent of levels of emotional exhaustion.

In the light of the complex interconnections among the predictor variables, the fourth step in data analysis employed multiple regression in order to test for the effect of conservative belief separately on emotional exhaustion and on satisfaction in ministry after taking into account the effects of personal factors, psychological factors, contextual factors, and experience factors. The two regression models (on the Scale of Emotional Exhaustion in Ministry and on the Satisfaction in Ministry Scale) are presented in Tables 8, 9. In these tables the first column carries the correlation coefficients brought forward from Table 7 for comparative purposes. Then the following columns present the incremental development of the model with personal factors entered in step one, psychological factors in step two, contextual factors in step three, experience factors in step four, and finally theological factors in step five.

**Table 8:** Regression on Scale of Emotional Exhaustion in Ministry

	*r*	Model 1	Model 2	Model 3	Model 4	Model 5
*Personal factors*						
Sex	−0.00	−0.01	−0.04	−0.06*	−0.09**	−0.10**
Age	−0.17***	−0.16***	−0.09**	−0.09**	−0.05	−0.06
*Psychological factors*						
Extraversion	−0.18***		−0.06*	−0.06*	−0.06*	−0.06
Neuroticism	0.56***		0.54***	0.54***	0.54***	0.54***
*Contextual factors*						
Married	−0.09**			−0.08*	−0.07*	−0.07*
Number of churches	0.00			−0.01	−0.02	−0.01
*Experience factors*						
Years in ministry	−0.11***				−0.08*	−0.09
Years in post	−0.06				−0.00	−0.01
*Theological factors*						
Conservative belief	−0.06					−0.06
*R*^2^		0.02	0.33	0.34	0.34	0.34
∆		0.02***	0.31***	0.01*	0.00	0.00

*p < .05, **p < .01, ***p < .001

**Table 9:** Regression on Satisfaction in Ministry Scale

	*r*	Model 1	Model 2	Model 3	Model 4	Model 5
*Personal factors*						
Sex	−0.02	−0.02	−0.01	−0.01	−0.00	0.02
Age	0.06	0.07	0.02	0.03	0.03	0.04
*Psychological factors*						
Extraversion	0.28***		0.19***	0.19***	0.18***	0.16***
Neuroticism	−0.42***		−0.39***	−0.39***	−0.38***	−0.38***
*Contextual factors*						
Married	0.04			0.01	0.01	0.01
Number of churches	−0.08*			−0.07*	−0.07*	−0.08**
*Experience factors*						
Years in ministry	0.04				−0.00	0.02
Years in post	0.09**				0.06	0.07*
*Theological factors*						
Conservative belief	0.19***					0.18***
*R*^2^		0.01	0.22	0.22	0.23	0.26
∆		0.01	0.21***	0.00	0.00	0.03***

*p < .05, **p < .01, ***p < .001

In terms of emotional exhaustion in ministry, model five (Table 8) demonstrates that the strongest predictor is provided by neuroticism scores. When neuroticism scores are taken into account additional variance is accounted for by three other variables. First, the negative association with sex shows that female ministers recorded lower levels of emotional exhaustion. Second, the negative association with married status shows that single ministers recorded higher levels of emotional exhaustion. Third, the negative association with years in ministry shows an inverse relationship between years served in ministry and levels of emotional exhaustion. What is revealing in this finding is that years served in ministry removed the significant effect of age. This adds further support for the view that the correlation (Table 6) reported between age and emotional exhaustion reflected a cohort effect rather than an ageing effect.

In terms of satisfaction in ministry, model five (Table 9) demonstrates that the strongest predictor is provided by neuroticism scores. When neuroticism scores are taken into account additional variance is accounted for by four other variables. First, the positive association with extraversion shows that extraverts record higher satisfaction in ministry than introverts. Second, the negative association with number of churches shows that as the number of churches increases, so satisfaction in ministry decreases. Third, the positive association with years in post shows that satisfaction in ministry increases with the time spent in a post. Fourth, the positive association with conservative belief shows that ministers who espouse conservative beliefs also record higher satisfaction in ministry.

### Limitations

The first limitation with the present study is that it was conducted on ageing data collected in 2008. It is nonetheless the most recent data on this theme available for Methodist circuit ministers in Great Britain. The study was conducted in 2008 to replicate and expand an earlier study conducted in 1997, with the measure of work-related psychological wellbeing added for the first time in 2008. A further replication is needed to bring the investigation of the work-related psychological wellbeing of Methodist ministers in Britain up to date. The second limitation with the present study is the way in which it has employed cross-sectional data to explore a causal theory. While the data can be properly employed to demonstrate that the evidence is *consistent* with causal theory, it cannot be taken to demonstrate causation. A different research design is required for that purpose in the sense of a longitudinal study.

## Conclusion

The present study was designed to assess the association between conservative Christian belief and work-related psychological wellbeing among Methodist circuit ministers in Great Britain. The research question was operationalised through the Francis Burnout Inventory that differentiates between negative affect (measured in terms of emotional exhaustion in ministry) and positive affect (measured in terms of satisfaction in ministry). Together these two measures implement the balanced affect approach to work-related psychological wellbeing among religious professionals according to which high levels of positive affect have been shown to mitigate the deleterious consequences of negative affect. Analysis of data provided by 803 ministers allows the following three main conclusions to be drawn.

The first main conclusion is that, after taking into account the effects of personal factors (sex and age), psychological factors (extraversion and neuroticism), contextual factors (married and number of churches), and experience factors (years in ministry and years in current post), holding conservative Christian belief was associated with higher levels of positive affect (satisfaction in ministry) but independent of negative affect (emotional exhaustion in ministry). This finding is the core original contribution to knowledge made by the present paper. This finding is of importance both for theoretical and practical purposes.

From a theoretical perspective, this finding adds further validation for the view that positive affect and negative affect operate as semi-independent systems, as well as being highly and negatively correlated. The evidence from the present study is that conservative Christian belief is related to individual differences in positive affect, but unrelated to individual differences in negative affect. Although in a cross-sectional study it is not possible to demonstrate causality, causal theories can be advanced that are consistent with the observed data. Assuming that conservative Christian belief impacts wellbeing rather than assuming that wellbeing impacts conservative Christian belief, the following theoretical explanation may be of relevance. On the one hand, levels of emotional exhaustion in ministry may be beyond the control of the majority of ministers, and narratives about the nature of God may struggle to nuance the interpretation of the experience of emotional exhaustion. On the other hand, narratives about the nature of God may be more directly influential in shaping the distinctive priorities that ministers express in their work and the account that they give for shaping ministry in their preferred way. Those who hold conservative Christian belief may find it easier to imagine themselves functioning as the direct agent of their God in the daily practice of ministry. Further research is now needed to test this speculative theory.

From a practical perspective, and read against the rationale for the balanced affect approach to clergy work-related psychological wellbeing, enhanced positive affect serves to mitigate the deleterious consequences of negative affect. On this account, ministers who espouse conservative Christian belief may be significantly less prone to burnout than colleagues who eschew conservative Christian belief. Put another way, more liberal and more inclusive ministers may be more vulnerable to burnout.

The second main conclusion is that, when personal factors, psychological factors, contextual factors, experience factors, and theological factors (holding conservative Christian belief) are placed side-by-side, the strongest predictors of individual differences in both negative affect and positive affect are psychological factors. Neuroticism scores lead the way, showing a positive correlation with emotional exhaustion in ministry (*r* = 0.56, *p* < .001) and a negative correlation with satisfaction in ministry (*r* = -0.42, *p* < .001). Extraversion scores follow in second place, showing a positive correlation with satisfaction in ministry (*r* = 0.28, *p* < .001) and a negative correlation with emotional exhaustion in ministry (*r* = -0.18, *p* < .001). This finding is consistent with the broad consensus from previous studies that finds psychological factors account for more variance in burnout measures than personal, contextual, or theological factors (Francis, 2018). The practical implication from this consistent finding is that routine psychological profiling, using appropriate personality measures, could predict ministers most susceptible to poor work-related psychological wellbeing. Such prediction could help to target intervention and therapeutic strategies.

The third main conclusion arises from setting the scores of these Methodist circuit ministers serving in Great Britain recorded on the Scale of Satisfaction in Ministry and on the Emotional Exhaustion in Ministry Scale alongside the scores recorded on the same measures by other groups of clergy and religious professionals. Placed alongside data from the eight other clergy studies cited in Table 5, Methodist circuit ministers occupied the following positions. Methodist ministers were among the two highest scoring groups of clergy in terms of emotional exhaustion in ministry, placed alongside Church of England clergy. Methodist ministers were among the two lowest scoring groups of clergy in terms of satisfaction in ministry, again placed alongside Church of England clergy. This positioning in the league table of poor work-related psychological wellbeing may be worth taking seriously.

## Data Availability

Data are available from the corresponding author upon reasonable request.
